# Managing an Advanced Heart Failure Patient at Home With a Long-Term Continuous Intravenous Furosemide Infusion

**DOI:** 10.1016/j.jaccas.2024.102611

**Published:** 2024-10-02

**Authors:** Julie G. Chirnside, Catherine J. Malone, Joanne G. Scott, Ian G. Crozier

**Affiliations:** a Cardio-respiratory Integrated Specialist Services, Te Whatu Ora Health NZ, Christchurch, New Zealand; b Department of Cardiology, Christchurch Hospital, Te Whatu Ora Health NZ, Christchurch, New Zealand

**Keywords:** cost-savings, diuretics, furosemide, heart failure, home, infusion, intravenous, nursing, palliative, quality of life

## Abstract

**Objective:**

To describe a simple therapeutic intervention for persistent congestion in an advanced heart failure patient using a continuous intravenous furosemide infusion in the home setting with a non-powered elastomeric pump that can be managed by the patient.

**Key Steps:**

Patient selected as a known intravenous furosemide responder with a supportive home environment. Shared care decision making with patient, cardiologist, heart failure nurse practitioner, palliative care physician, and general practitioner. Initiate this method of furosemide administration as an inpatient to test feasibility and determine choice of intravenous access. Communication links among lead prescriber, patient, hospital pharmacy, community nursing coordinator, patient’s primary care practitioners. Community intravenous nursing support to initially change pumps, maintain intravenous line and site dressing, blood draw, teach patient to self-manage the prefilled pump changes. Baxter for compounding prefilled pumps.

**Potential Pitfalls:**

Permanent intravenous lines carry the risk of thrombosis, migration, and infection. High dose furosemide may cause ototoxicity, renal injury, electrolyte disturbance and hypotension.

**Take-Home Messages:**

Persistent congestion from chronic heart failure can be safely managed in the home with a continuous high dose intravenous furosemide infusion. In our experience, the use of elastomeric pumps has provided a simple, safe, and effective method of delivering intravenous diuretic therapy when coordinated by heart failure nurses.

## Graphical Abstract

**Figure:**
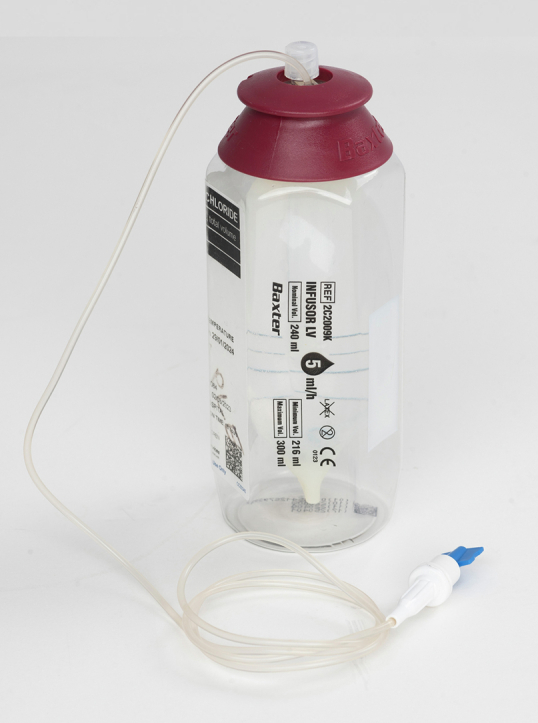

Living with heart failure (HF) is associated with high personal cost due to worsening physical symptoms, negative impacts on psychological and spiritual health, and social restriction for the patient and family.^1^ The HF trajectory is uncertain with the possibility of sudden death or progressive functional decline often resulting in multiple hospitalizations for HF or commonly associated health conditions. This incurs enormous health system costs. Guidelines for a palliative approach are available but reducing symptom burden and hospitalizations remain challenging. Guidelines suggest diuretic doses to “relieve congestion, improve symptoms and prevent worsening HF.”^2^ A total daily maximum dose of furosemide 400 to 600 mg has been recommended.^3^

**Take-Home Messages**

• The experience of a single patient shows that persistent congestion from chronic HF can be safely managed at home with a continuous high-dose IV furosemide infusion.
• In our experience, the use of elastomeric pumps has provided a simple, safe, and effective method of delivering IV diuretic therapy when coordinated by HF nurses.

The management of HF with preserved ejection fraction relies on treating underlying conditions, and decongestion with loop diuretics as first line. The loop diuretic, furosemide, when administered by the oral route provides 10% to 100% bio-availability, and therefore an unreliable effect. When gut edema is present, as is common in severe HF, oral diuretics are less effective, and doses escalate. In recent years, options for management of persistent congestion in end-stage HF include subcutaneous furosemide infusions in the community, hospice, or home setting^4^ with dose ranges up to 300 mg in 24 hours,^5^ and intravenous (IV) loop diuretics for a more immediate and consistent response. Administering IV diuretics in a day-stay lounge,^6^ and in small home-based studies,^7^ has been shown to be safe and reduce costs.^6^ The maximum dose of diuretics remains determined by individual clinicians.

In 2012, our HF service began using elastomeric pumps (Baxter International, Df2-1w) that are simple, portable, and do not require a power source to deliver continuous IV furosemide in a domiciliary setting. We manually filled the pumps. An internal audit of the first 3 years of practice for 27 patients showed most people’s symptoms improved, and all patients preferred having the infusion at home. Doses ranged from 250 to 2,000 mg over 24 hours. There were no significant adverse events. At the time of writing, more than 50 patients have received home infusions. The unique aspect in this report is the infusion pump is prefilled and managed by the patient with nursing support from the community and HF nurse practitioner (NP). IV furosemide is the patient’s preferred route of administration for effective symptom control.

## Case Summary

A 79-year-old woman with a history of treated ischemic heart disease, atrial tachycardias, and fibrillation with atrioventricular nodal ablation and permanent pacemaker; preserved left ventricular function, and comorbidities including chronic kidney disease estimated glomerular filtration rate 35 to 42 mL/min/1.73 m^2^, had multiple hospitalizations for predominant right HF over the course of 1 year. Treatment options were limited because of medication intolerances including empagliflozin, metolazone, spironolactone, eplerenone, and acetazolamide. Diuretic doses were escalated through the year, eventually adding IV 80 mg bolus furosemide twice weekly at her primary practice, but this was ineffective. The patient was referred from primary practice to the community palliative care service, and daily subcutaneous furosemide was added to oral therapy. The patient was readmitted to hospital later that month with worsening dyspnea, orthopnea, angina, nausea, and fluid overload.

The patient was breathless at rest, respiratory rate 24 breaths/min. Blood pressure 105/62 mm Hg, oxygen saturations 98% on air, heart rate 76 beats/min in a ventricular paced rhythm with underlying atrial fibrillation. Heart sounds dual with an ejection systolic murmur at the left sternal edge. Recumbent at 45 °jugular venous pressure elevated +5 cm, positive hepato-jugular reflux, grossly distended abdomen with palpable liver edge, without lower limb or sacral edema. Bilateral inspiratory crepitations at lung bases. The chest x-ray revealed venous congestion. Abnormal biochemistry included K+ 3.4 mmol/L, urea 8.2 mmol/L, estimated glomerular filtration rate 53 mL/min/1.73 m^2^, gamma-glutamyl transferase 62 U/L. No iron deficiency or anemia. B-type natriuretic peptide 24 pmol/L, noted to never have elevated above 36 pmol/L with any HF admission. Differential diagnoses were considered and ruled out. Cardiac medications were oral furosemide 1,000 mg twice daily and subcutaneous furosemide 80 mg daily, isosorbide mononitrate 120 mg daily, quinapril 2.5 mg daily, clopidogrel 75 mg daily, rosuvastatin 10 mg daily, dabigatran 75 mg twice a day, and glyceryl trinitrate as required.

Oral and subcutaneous furosemide were discontinued and 1,500 mg IV furosemide over 24 hours was administered for 2 days via peripheral access. The medical team and HF NP discussed with the patient the option of continuing the infusion at home as a palliative strategy using 120 mL SV elastomeric pumps every 24 hours.

**Figure 1:**
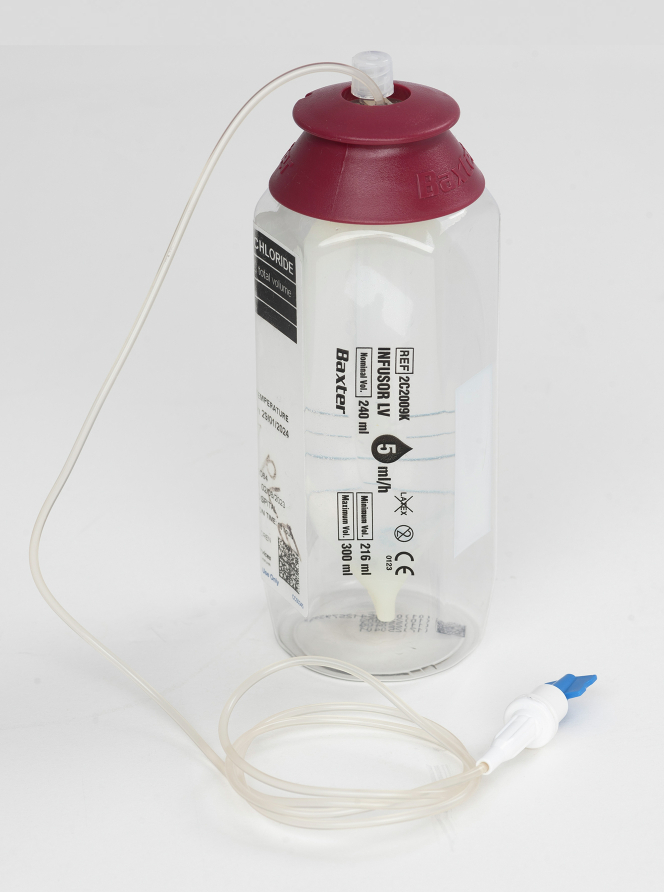
Device Used for the Delivery of Continuous High-Dose Intravenous Furosemide A 240-mL LV5 elastomeric pump delivers intravenous therapy at 5 mL/h over 48 hours.

The home infusion was successful with patient-reported symptomatic, functional capacity, and quality-of-life benefits, and without rehospitalization. As safety was demonstrated, the infusion was made more manageable by compounding furosemide into prefilled 240-mL LV5 pumps (Figure 1) to last 48 hours. The infusion has continued for 17 months, with the patient managing the pump changes supported by the community nurses and HF NP.

Total costs including hospital admissions for the 6 months before the infusion commencing were NZ$29,753.79, and for the 6 months following NZ$2,756.42.

## Procedural Steps

**Figure 2:**
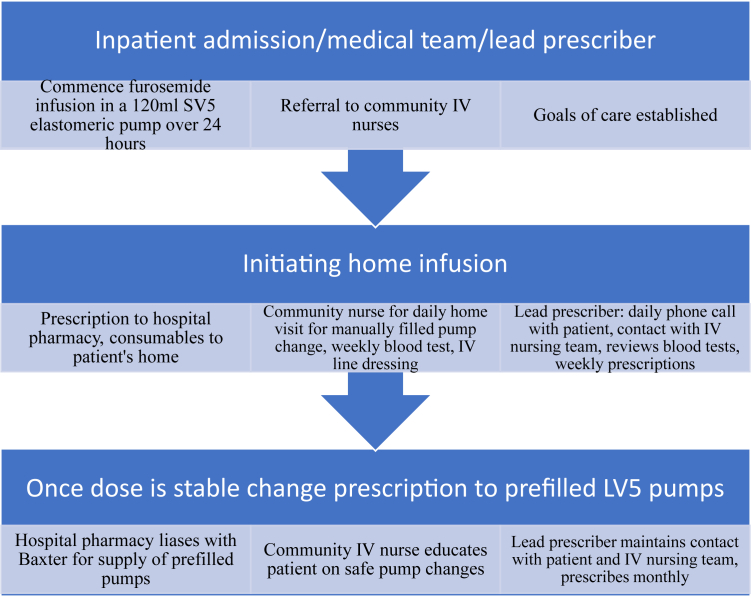
Process of Initiating and Maintaining a Continuous IV Furosemide Infusion in the Home Using Elastomeric Pumps Initiating and assessing the safety and feasibility for a patient using continuous intravenous furosemide infusion at home requires frequent oversight in the early stages. Regular oversight continues at reduced frequency in the maintenance phase and may allow the patient to manage prefilled pumps. IV = intravenous.
• Establish lead prescriber (Figure 3).

• Verbal explanation to the patient of potential benefits of the infusion, and harms and complications that can occur with high-dose furosemide and central lines. Verbal consent was obtained for the infusion and plan.
• Written consent obtained for the peripherally inserted central catheter (PICC) and later chest inserted central catheter (CICC).
• Written prescription for “1,000 mg furosemide (10 mg/mL = 100 mL) and 20 mL normal saline in a 120-mL SV elastomeric pump to infuse via PICC line over 24 hours (5 mL/h).”
• Two registered nurses in the cardiac ward manually filled the pumps using an aseptic technique.
  ○ Draw up 100 mL (10 mg/mL) furosemide in two 50-mL luer-lock syringes
  ○ Draw up 20 mL normal saline in one 20-mL luer-lock syringe
  ○ Remove the bung from the insertion port of the SV 120-mL pump
  ○ Attach and depress 1 syringe at a time to the insertion port on the pump and fill the balloon to a total of 120 mL
  ○ Screw the bung back on the insertion port
  ○ Remove the blue bung from the end of the tubing to prime the line (solution appears at the end of the line)
  ○ Reattach the blue bung to the tubing once primed
  ○ Check the prescription and patient identification
  ○ Remove the bung from the primed tubing and connect to the PICC using an aseptic technique
• Infusion initiated the day before hospital discharge to ascertain effect and confirm that patient wished to continue at home (Figure 2).

**Figure 3:**
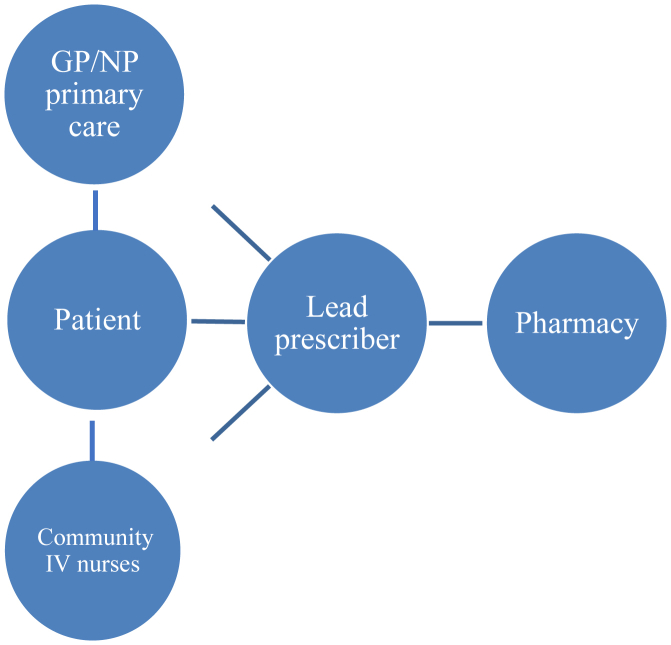
Model of Care for IV Furosemide Infusions in the Home Setting The lead prescriber coordinates IV furosemide infusions for community patients with advanced heart failure. IV = intravenous.
• Establish dry weight target before discharge.
• Acute and advance care plans and goals of care established and documented.
• Referred to the community IV nursing team for
  ○ daily home visits to manually fill and change the pump
  ○ monitor the PICC for signs of infection, thrombosis, or migration
  ○ discuss any concerns with HF NP
  ○ weekly line dressings and blood draw for renal function and electrolytes
• HF NP as lead prescriber’s role:
  ○ daily conversation with the patient to monitor weight, symptoms, and side effects
  ○ liaise with the community IV nursing team coordinator
  ○ review blood tests
  ○ write ongoing prescriptions
  ○ coordinate with pharmacy for supply of consumables to patient’s home
• Once the dose proved effective, the prescription changed to “2,000 mg furosemide (10 mg/mL = 200 mL) with 40 mL normal saline in a 240-mL LV5 prefilled pump over 48 hours (5 mL/h) via CICC.”
  ○ prescriptions to hospital pharmacy
  ○ pharmacy arranged for infusions to be compounded by Baxter into prefilled LV5 pumps with stability of 9 days at room temperature
  ○ community nurses educated the patient to safely change the pump
  ○ patient contact with the HF NP reduced to monthly, or as needed, to check status before prescribing a further month’s prescription

## Potential Pitfalls

Although the dose of furosemide and method of administration goes beyond published guidelines, care was taken to monitor for adverse effects such as renal injury, electrolyte disturbance, ototoxicity, hypotension, and central line complications such as migration, infection, and thrombosis. The patient saw a nurse every day for the first few weeks, then alternate days, then weekly. Concerns for PICC migration were raised after 4 months by the nurses. A chest x-ray was ordered and PICC was replaced with a CICC. No further issues have arisen.

Patient selection is important when considering this option, as they require a supportive home environment, be known to respond well to IV furosemide infusions in hospital, and have goals of care established.

## Conclusions

Continuous IV furosemide infusion at home using elastomeric pumps is a novel strategy for improving HF care for those with advanced disease requiring higher doses than what is delivered in subcutaneous infusions. Identifying suitable patients for home infusions may reduce the economic burden of HF by reducing length of stay and avoiding HF hospital admissions. We report a case of a patient with advanced HF receiving continuous IV furosemide at home for 17 months without further hospitalization. This has resulted in improved patient well-being and significant cost-savings.

## Funding Support and Author Disclosures

The device used is supplied through our hospital pharmacy’s sterile unit with the infusions compounded offsite by Baxter. Permission has been obtained to use the image of the device. No financial remuneration has been received by industry or other source. The authors have reported that they have no relationships relevant to the contents of this paper to disclose.

## References

[1] Rogers J.G., Patel C.B., Mentz R.J. (2017). Palliative care in heart failure: the PAL-HF randomized, controlled clinical trial. J Am Coll Cardiol.

[2] Heidenreich P.A., Bozkurt B., Aguilar D. (2022). 2022 AHA/ACC/HFSA Guideline for the management of heart failure: a report of the American College of Cardiology/American Heart Association Joint Committee on clinical practice guidelines.

[3] Mullens W., Damman K., Harjola V.-P. (2019). The use of diuretics in heart failure with congestion—a position statement from the Heart Failure Association of the European Society of Cardiology. ESC Heart Fail.

[4] Zacharias H., Raw J., Nunn A. (2011). Is there a role for subcutaneous furosemide in the community and hospice management of end-stage heart failure?. Palliat Med.

[5] Brown A., Westley K., Robson J. (2022). Furosemide in end-stage heart failure: community subcutaneous infusions. BMJ Support Palliat Care.

[6] Ioannou A., Browne T., Jordan S. (2020). Diuretic lounge and impact on hospital admissions for treatment of decompensated heart failure. QJM.

[7] Payne D. (2021). Intravenous diuretic administration in the home environment. Br J Community Nurs.

